# Locally Acquired Plasmodium falciparum Malaria in a Non-endemic Region of Lebanon: A Case Report

**DOI:** 10.7759/cureus.97435

**Published:** 2025-11-21

**Authors:** Sadek Hashem, Tahsine Mahfouz, Atika Berry, Hassan Salameh, Mahdi Tarhini

**Affiliations:** 1 Medical Laboratory Department, Sheikh Ragheb Harb University Hospital, Nabatieh, LBN; 2 Infection Control Department, Sheikh Ragheb Harb University Hospital, Nabatieh, LBN; 3 Preventive Medicine Department, Ministry of Public Health (MOPH), Beirut, LBN

**Keywords:** autochthonous malaria, locally acquired malaria, malaria in lebanon, non-endemic region, plasmodium falciparum

## Abstract

Malaria is a parasitic disease caused by several *Plasmodium* species, transmitted through the bite of infected *Anopheles mosquitoes,* and capable of causing mild to life-threatening illness. In non-endemic areas, sporadic cases present a significant diagnostic challenge, where delay in recognition can lead to severe outcomes and death. In this case report, we present a rare case of locally acquired *P. falciparum* malaria in Lebanon, in a 70-year-old Lebanese female with no history of travel to endemic regions, who presented with intermittent fever, chills, headache, nausea, and abdominal pain. Laboratory tests showed anemia, thrombocytopenia, and leukopenia. Microscopic investigations on peripheral blood smear revealed intraerythrocytic ring forms, and a rapid diagnostic test (RDT) confirmed *P. falciparum* infection. Given the patient’s age and high parasitemia, she was treated with intravenous artesunate, followed by oral artemether-lumefantrine, and subsequently made a full recovery. The absence of conventional exposure routes, such as travel, blood transfusion, or organ transplant, makes this case a significant diagnostic and public health challenge. This case highlights the need for sustained malaria surveillance and heightened awareness among clinicians in historically non-endemic areas.

## Introduction

Malaria, a mosquito-borne infection caused by several *Plasmodium *species, remains a major global health challenge. Symptoms vary from mild manifestations such as intermittent fever, chills, headache, and fatigue to life-threatening complications, including severe anemia, thrombocytopenia, and multi-organ dysfunction. *Plasmodium falciparum* is responsible for the majority of severe disease and deaths worldwide [1]. According to the World Health Organization’s 2024 Malaria Report, an estimated 263 million cases occurred globally in 2023, resulting in approximately 597,000 deaths [1]. Notably, the Eastern Mediterranean Region has experienced a 57% rise in reported cases since 2021 [1]. In non-endemic countries, malaria is typically suspected only among travelers returning from endemic areas. However, sporadic cases occasionally occur in the absence of travel history, known as autochthonous (locally acquired) malaria. Such cases pose diagnostic and epidemiological challenges due to delayed recognition and treatment. Transmission in non-endemic areas may occur via unconventional routes such as ‘airport malaria,’ nosocomial transmission, or rare cryptic vector-borne infections. Lebanon was declared free of indigenous malaria transmission in 1963 [2]. Since then, all confirmed cases have been presumed imported. In 2012, Lebanon recorded 115 malaria cases, half attributed to *P. falciparum*. A case reported in Sidon in 2019 by the Ministry of Public Health lacked sufficient epidemiological data to determine whether it was imported or locally acquired [3]. In December 2024, Abou Hamdan et al. documented Lebanon’s first confirmed locally transmitted case in a 27-year-old woman with no travel history but a plausible nosocomial exposure [4]. In this study, we describe a confirmed case of locally transmitted malaria in Lebanon in a patient without a recent travel history to endemic areas. Our objective is to emphasize the critical need for enhanced surveillance and clinician awareness regarding malaria in non-endemic regions.

## Case presentation

**Figure 1:**
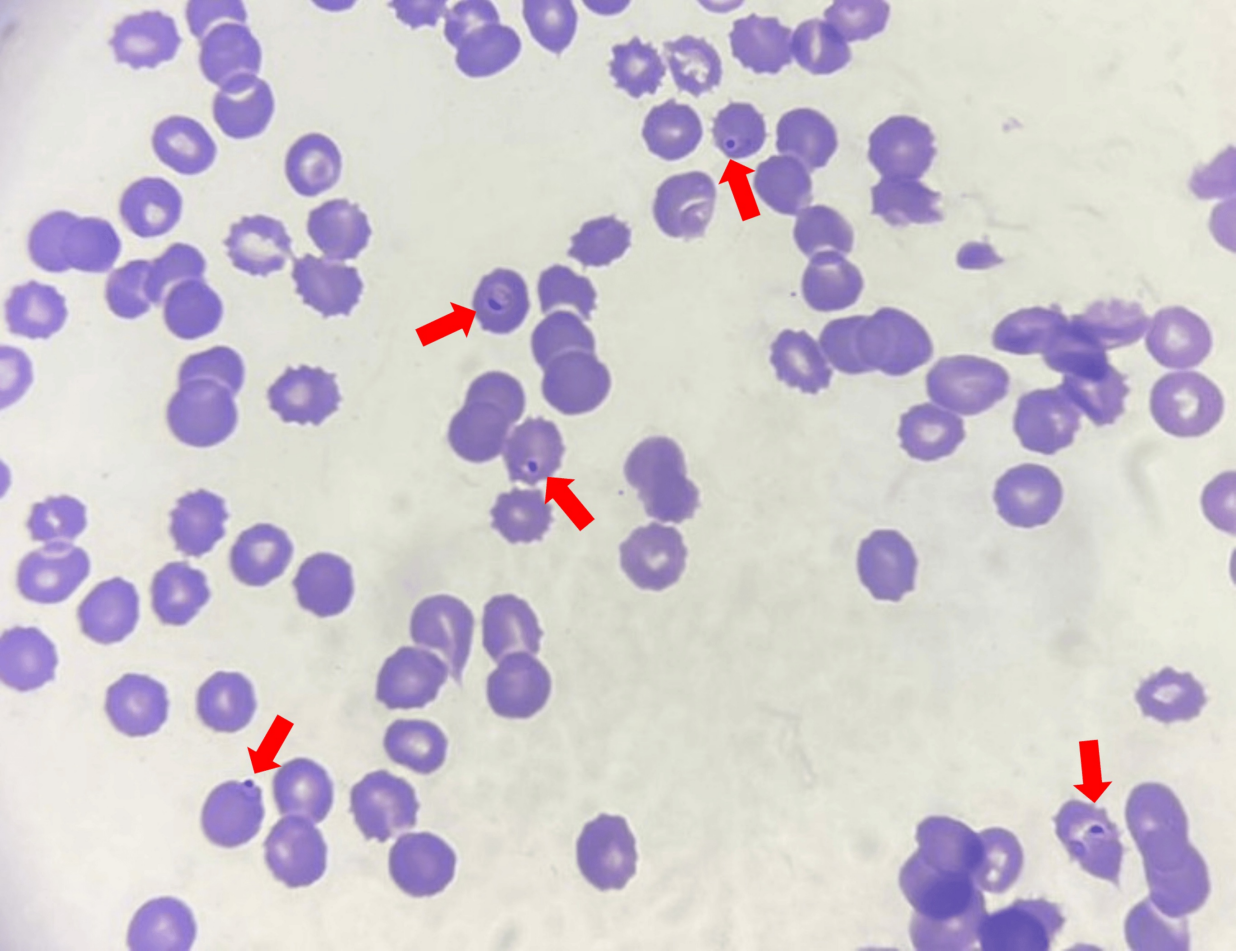
Peripheral blood smear (eosin methylene blue stain, 100x oil immersion) showing intraerythrocytic ring forms of P. falciparum (red arrows)

**Figure 2:**
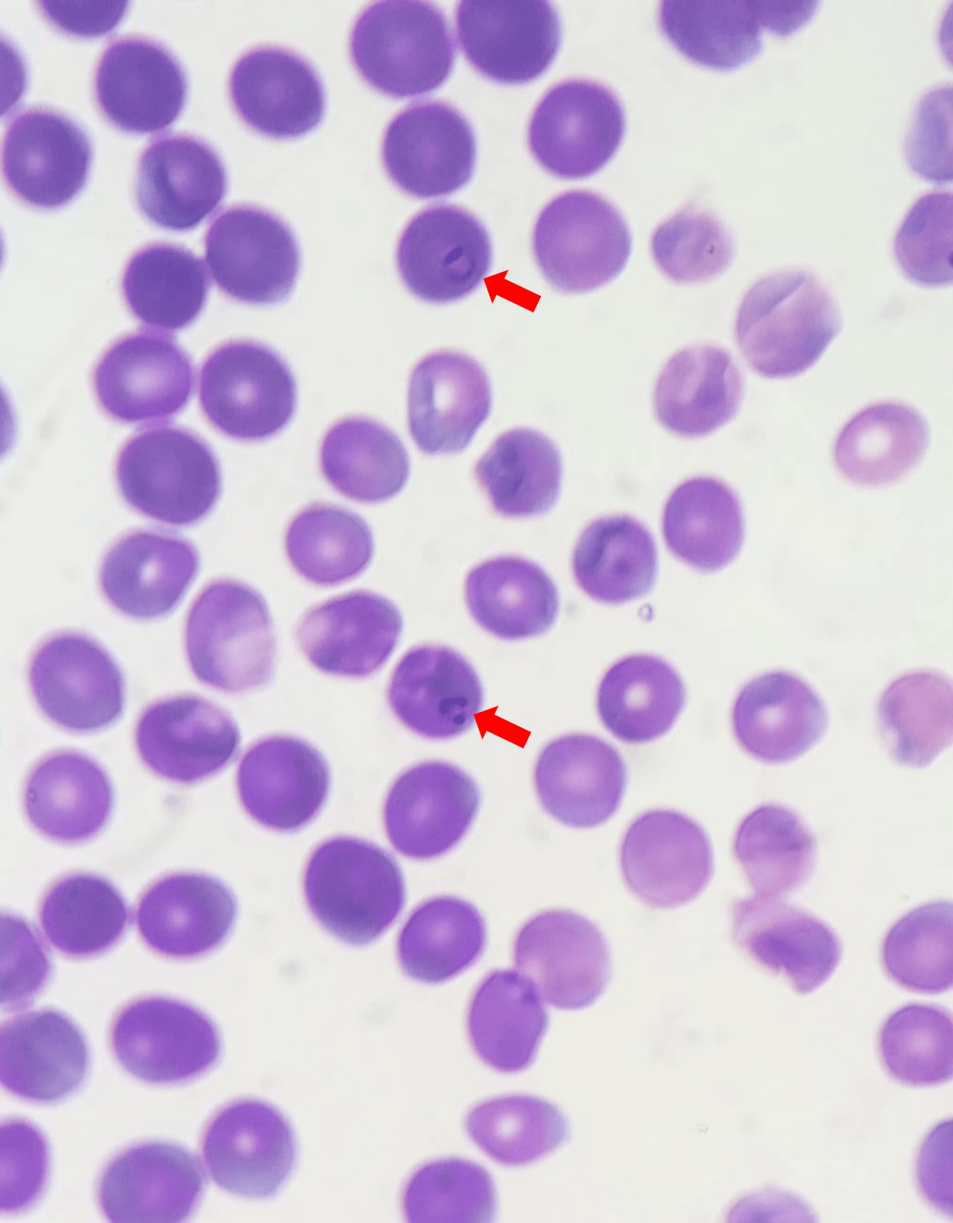
P. falciparum ring forms in a second microscopic field (eosin methylene blue stain, 100x oil immersion) (red arrows)

**Figure 3:**
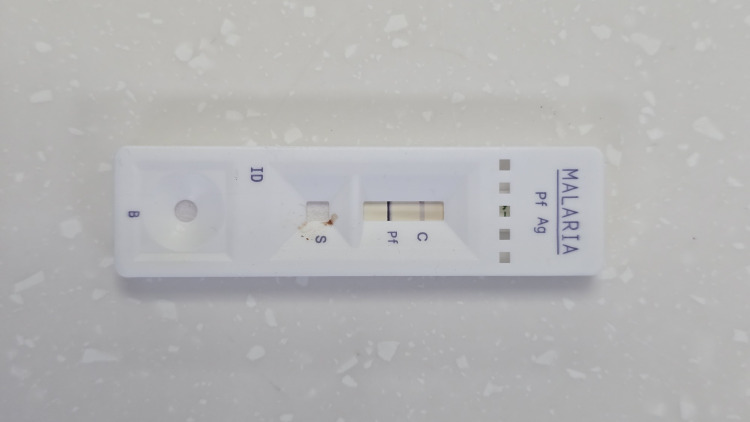
Rapid diagnostic test (RDT) showing a positive result for P. falciparum antigen

**Figure 4:**
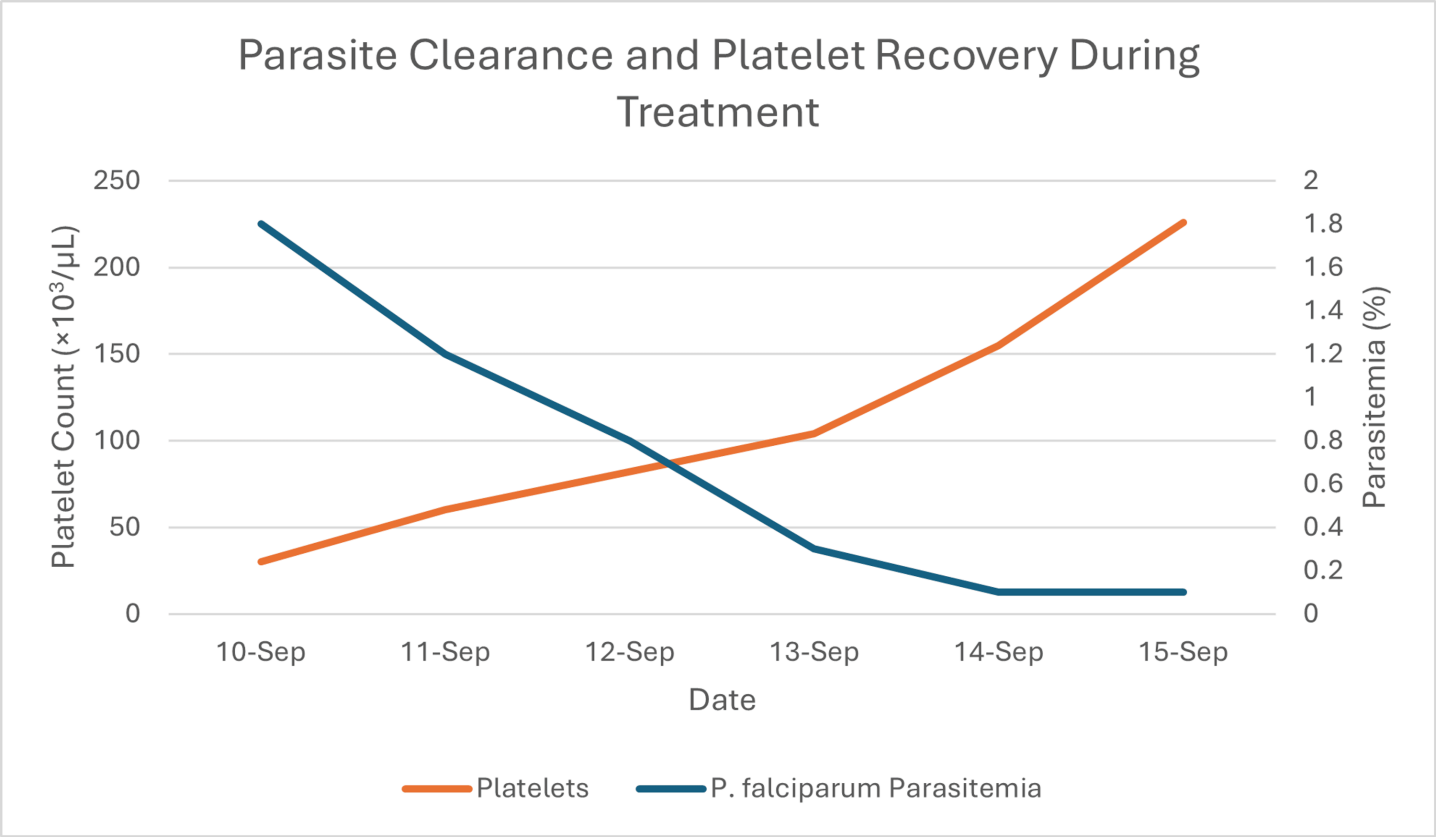
Time course of parasite clearance and platelet recovery This graph illustrates the inverse relationship between parasite load and platelet count, beginning from the patient's admission on September 10. The right axis shows the *P. falciparum* parasitemia (blue line), which declined from 1.8% on admission to 0.1% by September 14, indicating successful anti-malarial treatment. The left axis shows the corresponding resolution of severe thrombocytopenia (red line), with the platelet count recovering from a critical low of 30×10³/µL on admission to 226×10³/µL by September 15.

**Figure 5:**
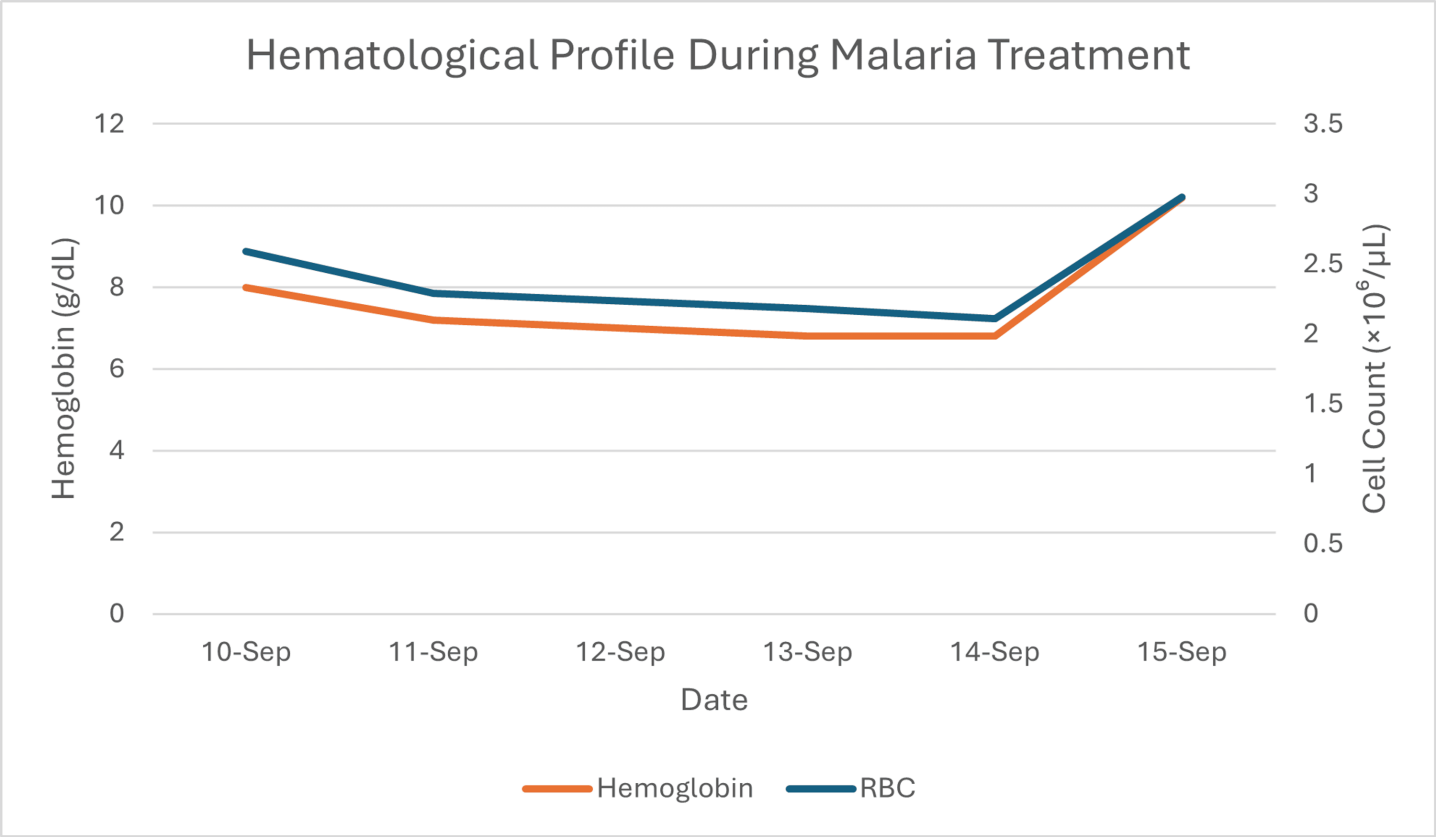
Hematological profile during malaria treatment This graph shows the patient's hemoglobin (red line, left axis) and red blood cell (RBC) count (blue line, right axis) from admission on September 10. The patient presented with anemia (8.0 g/dL Hgb, 2.59×10⁶/µL RBC), which initially worsened, consistent with malaria-induced hemolysis. The sharp, concurrent rise in both hemoglobin (to 10.2 g/dL) and RBCs (to 2.98×10⁶/µL) on September 15 is indicative of a blood transfusion.

**Figure 6:**
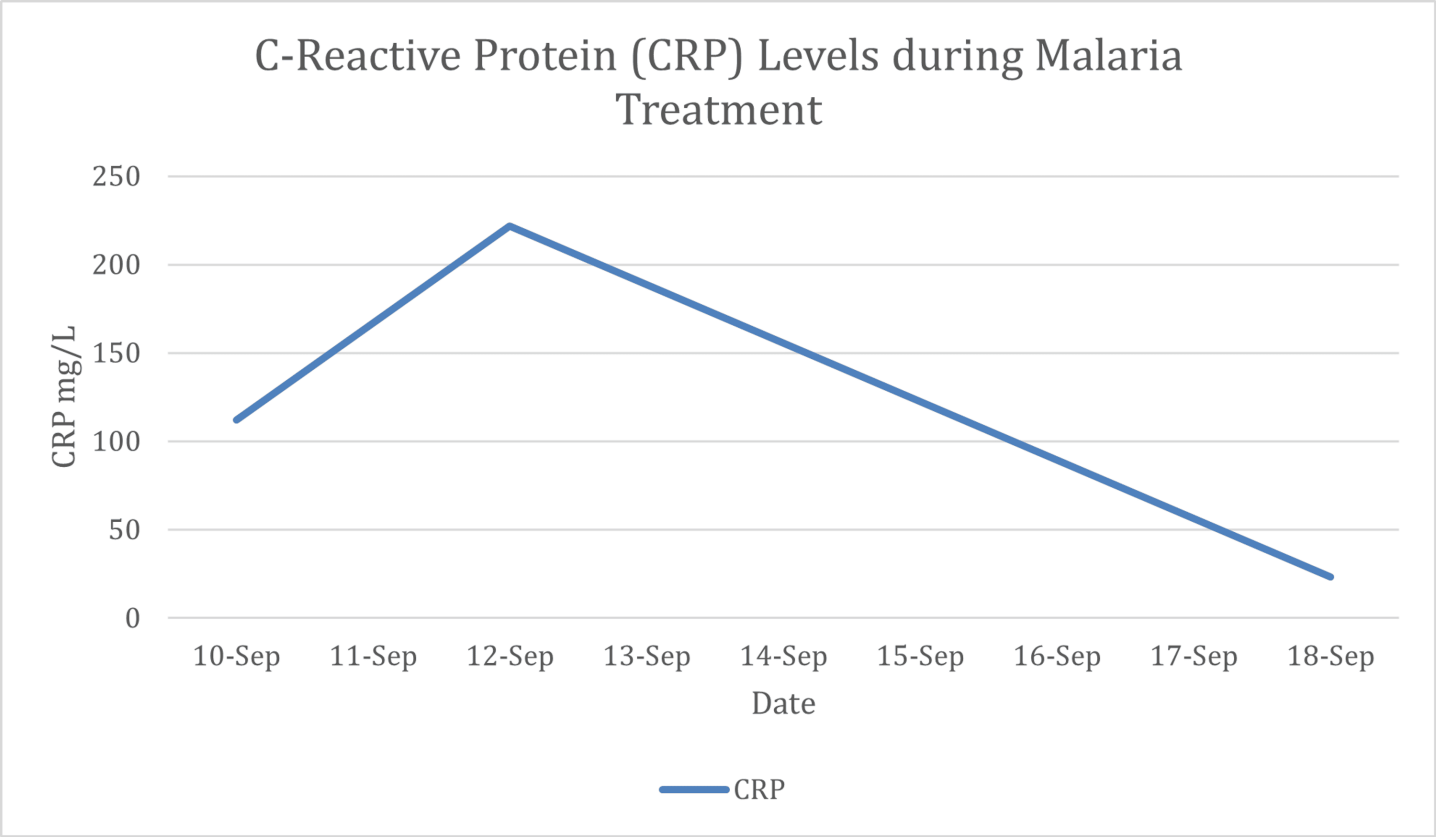
Time course of C-reactive protein (CRP) levels This graph illustrates the patient's acute inflammatory response. CRP was highly elevated at 112 mg/L on admission (September 10). The level spiked to 222 mg/L on September 12, likely representing a transient inflammatory surge in response to parasitic lysis after treatment initiation. This was followed by a marked decline to 23.3 mg/L by September 18, confirming a successful therapeutic response and the resolution of inflammation.

**Figure 7:**
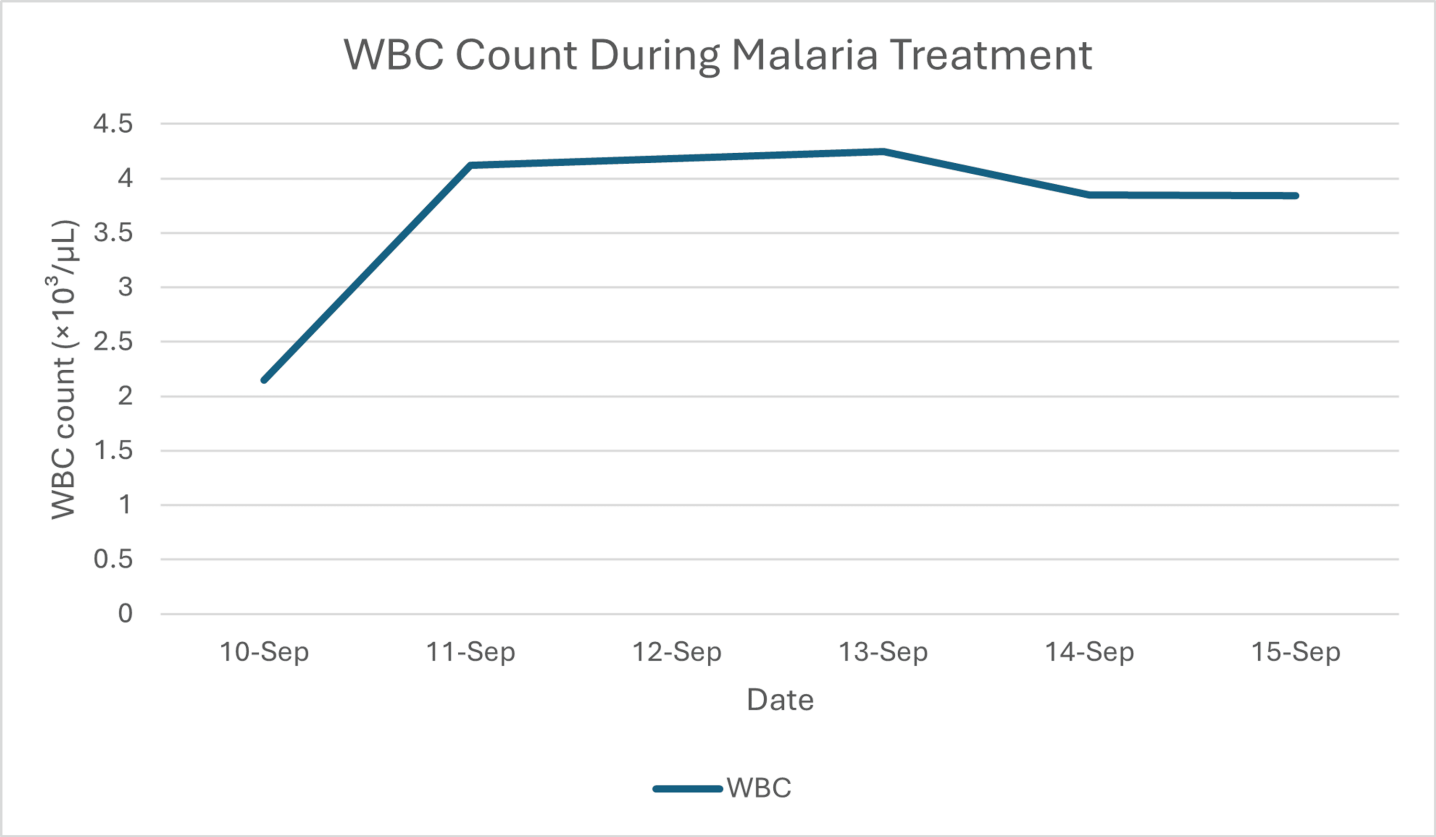
Time course of white blood cell (WBC) count This graph details the resolution of the patient's leukopenia. The WBC count, which was 2.15×10³/µL on admission (September 10), rapidly recovered into the normal reference range by the following day (4.12×10³/µL) and stabilized, indicating a recovery of the patient's immune response.

 A 70-year-old Lebanese woman from Houla, Southern Lebanon, presented to Sheikh Ragheb Harb University Hospital Emergency Department in September 2025 with intermittent fever (38.7°C), chills, sweating, headache, nausea, and abdominal pain. Initial laboratory findings revealed severe anemia (Hb 8 g/dL), leukopenia (WBC 2,150/µL), profound thrombocytopenia (platelets 30,000/µL), and elevated C-reactive protein (CRP) and troponin (Table 1). A peripheral blood smear revealed intraerythrocytic ring forms (Figures 1, 2), and a rapid diagnostic test (RDT) confirmed *Plasmodium falciparum* infection (Figure 3). The peripheral blood smear was prepared, stained, and confirmed by an expert clinical microbiologist in the medical laboratory. The species identification of *P. falciparum* was based on the morphological findings of delicate, small ring-form trophozoites, including some cells with multiple parasites, which are characteristic features. The patient reported no international travel to endemic regions and had not received blood transfusions or organ transplants. She resided in Houla but had been living in Safad for the past year. She had window screens but no air conditioning. Her recent visit to Ouzai, Beirut, near the Al Ghadir River, one month prior to symptom onset, was considered a possible ecological exposure. A parasitemia level of 1.8% in a non-immune, elderly patient is considered a high-risk indicator for rapid progression; therefore, immediate initiation of intravenous artesunate was clinically warranted to rapidly reduce the parasite burden. Intravenous artesunate therapy was initiated with a dose of 180 mg at zero, 12, and 24 hours, followed by 180 mg daily doses for three days. Subsequently, oral artemether-lumefantrine (Coartem) at a dose of 80 mg/480 mg two times a day was administered [5]. The patient's clinical and laboratory parameters showed marked improvement following the initiation of treatment, as detailed in the time-course graphs (Figures 4-7). Her *P. falciparum* parasitemia was successfully cleared, declining from 1.8% on admission to 0.1% (Figure 4). Concurrently, her severe thrombocytopenia resolved, with the platelet count recovering from 30×10^3^/µL to 226×10^3^/µL in the same period (Figure 4). Her leukopenia also resolved, with the WBC count rising from 2.15×10^3^/µL to 4.12×10^3^/µL (Figure 7). The patient's acute inflammatory response, indicated by a CRP of 112 mg/L, spiked to 222 mg/L before declining sharply to 23.3 mg/L. This temporary CRP spike likely represents an acute inflammatory surge, distinct from the initial infection, and was possibly triggered by the mass lysis of parasites after treatment began (Figure 6). Her anemia was stabilized with a blood transfusion, as shown in Figure 5. All symptoms resolved, and she was discharged in stable condition.

**Table 1:** Laboratory results on admission MCV: mean corpuscular volume; MCH: mean corpuscular hemoglobin; MCHC: mean corpuscular hemoglobin concentration; RDW-CV: red cell distribution width-coefficient of variation; CRP: C-reactive protein

Parameter	Value	Reference Range	Unit
WBC	2.15	4 - 11	×10^3^/µL
RBC	2.59	4.20 - 6	×10^6^/µL
Hemoglobin	8	12 - 16	g/dl
Hematocrit	23.8	36 - 46	%
Platelets	30	150 - 400	×10^3^/µL
MCV	94.4	80 - 100	FL
MCH	33.5	27 - 33	pg
MCHC	35.5	32 - 36	g/dl
RDW-CV	13.9	11 - 15	%
Neutrophil Percent	67.8	40 - 70	%
Lymphocyte Percent	22.3	20 - 40	%
Monocyte Percent	8.3	2 - 8	%
Eosinophil Percent	0.2	1 - 3	%
Basophil Percent	1.4	0 - 1	%
Urine Culture	No Growth After 48 Hours	No Growth After 48 Hours	
CRP	112	0 - 10	mg/l
Creatinine	0.83	0.44 - 1	mg/dl
Troponin	36.9	Negative <19 Grey Zone 19 - 100 Positive >= 100	ng/l
Malaria Thin Smear	Presence of *Plasmodium falciparum* ring 1.8%	0	%
Malaria Rapid Diagnostic Test	Positive	Negative	

## Discussion

This case of locally acquired *P. falciparum* malaria in Lebanon, a country free of endemic transmission for decades, is a significant public health alert. Identifying the precise location of infection is challenging; the patient, originally from Houla, had been living in Safad for the past year and also visited the Ouzai area near the Al Ghadir River one month prior to symptom onset. While the Al Ghadir River is a plausible source of exposure, the patient's movement between multiple locations makes it difficult to definitively pinpoint a single source of infection. This uncertainty highlights that the risk may not be isolated to one area and underscores the urgent need for new, widespread entomological surveillance to map the current distribution and density of *Anopheles* populations across all at-risk Lebanese regions. The threat is not hypothetical; historical and regional surveys have confirmed the presence of competent vectors in Lebanon, including *Anopheles* *claviger *and the primary regional vector, *Anopheles sacharovi* [6-8].

The re-emergence documented by this case is not an isolated phenomenon in the Mediterranean. Several other non-endemic European countries have recently reported sporadic, locally acquired cases. Greece, for example, has reported recurring locally acquired *P. vivax *cases and, notably, reported a locally acquired *P. falciparum* case in 2020 and two more in 2025 [9,10]. Italy recently reported its first locally acquired *P. vivax* case in decades, raising new concerns about reintroduction [11]. Similarly, France documented 117 locally acquired cases between 1995-2022, 88% of which were *P. falciparum* [12]. These events show a clear, though rare, regional vulnerability to malaria re-emergence.

This regional risk is amplified by local conditions. The case arises at a time when Lebanon's public health system is under unprecedented strain. The nation's ongoing economic crisis has resulted in critical shortages of medication and a significant "brain drain" of medical staff, severely compromising the capacity for robust infectious disease surveillance [13,14]. This vulnerability is compounded by climate change. The Mediterranean basin is recognized as a "hot spot" for climatic shifts, with rising temperatures and changing rainfall patterns creating more hospitable environments for disease vectors like *Anopheles* [15,16].

This convergence of a weakened health system, established vectors, and favorable environmental conditions creates a tangible risk for the reintroduction of malaria. The diagnostic blind spot for non-travelers, as seen in this case, is the greatest danger. Therefore, this case serves as a critical reminder to clinicians and healthcare workers: malaria must be included in the differential diagnosis for patients presenting with unexplained intermittent fever, chills, and thrombocytopenia, regardless of their travel history or location within Lebanon. Heightened clinical awareness and sustained national surveillance are essential to prevent further local transmission.

A limitation of this report is the lack of molecular characterization. This test was not prioritized clinically once the diagnosis was confirmed by gold standard microscopy, but we strongly recommend that PCR and sequencing be standard for any future public health investigation of suspected autochthonous cases.

## Conclusions

This case of locally acquired *P. falciparum* malaria, in a patient with no travel history and movement between multiple regions, confirms that the risk of local transmission in Lebanon, while rare, is present and may not be isolated to a single area. This re-emergence is not an isolated event but aligns with similar sporadic cases in other non-endemic Mediterranean countries. Therefore, this case serves as a critical alert for clinicians; malaria must be included in the differential diagnosis for any patient presenting with unexplained fever and thrombocytopenia, regardless of travel history. To prevent the re-establishment of malaria, we strongly recommend urgent public health actions: first, renewed nationwide entomological surveillance to map current *Anopheles *vector distribution and density; second, immediate epidemiological surveys and vector tracing to be conducted in the regions where the patient resided (Houla, Safad, and Ouzai near the Al Ghadir River) as a necessary precautionary measure to identify any potential local transmission hotspots; and third, strengthening national infectious disease surveillance to ensure any new case is rapidly detected and contained.
